# Supplementary material for: Identification of Freezing-Responsive microRNAs and Their Targets in Chinese Jujube by Small RNA and Degradome Sequencing
Source: Int J Mol Sci. 2026 Sep 4;27(17):7912. doi: 10.3390/ijms27177912 (PMC13566890; doi:10.3390/ijms27177912)
Supplement: Supplementary file 1 [file ijms-27-07912-s001.zip › Supplementary Material S2/novel_miR_4.pdf]

Provisional ID	:	novel_miR_4
Score total	:	30.7
Score for star read(s)	:	3.9
Score for read counts	:	28.7
Score for mfe	:	0.3
Score for randfold	:	-2.2
Score for cons. seed	:	
Total read count	:	68
Mature read count	:	65
Loop read count	:	0
Star read count	:	3

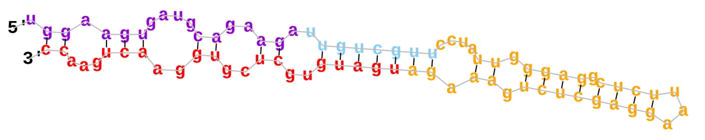

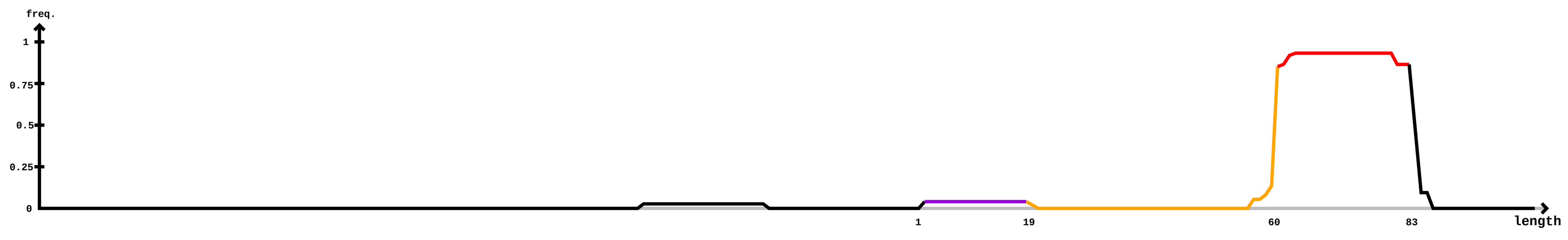

5'- uaguuuaacuauuuuuccuuguaauauuuugccuuauuauguuccaaaauauuugaucaacugcuuuucggcagaaauauacuaaaggcuugggugaaggccuucagaaagagagagagcaugaagaugaacaagauuaaugauuguggaagugaugcagaagauugucguuccuauugggaggcucuuaaggagcucugaaagaugaugugcucguggaacugaaccauuaguuuuguugaucggaaaa -3' obs

uaguuuaacuauuuuuccuuguaauauuuugccuuauuauguuccaaaauauuugaucaacugcuuuucggcagaaauauacuaaaggcuugggugaaggccuucagaaagagagagagcaugaagaugaacaagauuaaugauuguggaagugaugcagaagauugucguuccuauugggaggcucuuaaggagcucugaaagaugaugugcucguggaacugaaccauuaguuuuguugaucggaaaa exp

...........((((((..((((....))))....((((((((...............(((((....)))))......((.(((((((......))))))).)).........)))))))).(((.((((((((((((...((...(((....((..((..(((((((....((.((((.((((...))))))))).)).)))))))..))..))..)))..))))))))))))))).)))))))))

read	reads	mm	sample
gccuucagaaagagagagagU	2	1	LT-2
uggaagugaugAagaaga	3	1	LT-2
aagaugaugugcucguggaacuga	4	0	LT-2
gaugaugugcucguggaacuga	1	0	LT-3
gaugaugugcucguggaacugaacca	1	0	LT-2
augaugugcucguggaacugaaccauu	1	0	LT-3
augaugugcucguggaacugaaccauu	3	0	LT-2
ugaugugcucguggaacugaacc	1	0	LT-3
ugaugugcucguggaacugaCcc	1	1	LCK-2
ugaugugcucguggaacugaacc	1	0	LT-1
ugaugugcucguggaacugaacc	1	0	LT-2
ugaugugcucguggaacugaacc	24	0	LCK-1
ugaugugcucguggaacugaacc	23	0	LCK-2
ugaugugcucguggaacugaaccauu	2	0	LT-2
gaugugcucguggaacugaacc	1	0	LT-3
augugcucguggaacugaacc	4	0	LCK-3
ugugcucguggaacugaaccauu	1	0	LT-3
